# Annotated genome of the Atlantic dog whelk, *Nucella lapillus*

**DOI:** 10.1093/g3journal/jkaf182

**Published:** 2025-08-09

**Authors:** Meghan R Ford, Steven V Vollmer, Geoffrey C Trussell

**Affiliations:** Marine Science Center and Coastal Sustainability Institute, Northeastern University, Nahant, MA 01908, United States; Marine Science Center and Coastal Sustainability Institute, Northeastern University, Nahant, MA 01908, United States; Department of Biological Sciences, Florida Atlantic University, Boca Raton, FL 33431, United States; Marine Science Center and Coastal Sustainability Institute, Northeastern University, Nahant, MA 01908, United States

**Keywords:** Atlantic dog whelk, *Nucella*, genome assembly, annotation, mollusks, animalia

## Abstract

*Nucella lapillus* is an important player in rocky shore food chains and has been a focal organism of ecological and evolutionary studies for decades. Despite poor dispersal, they have a broad geographic range, which makes them an ideal species to examine isolation by distance and selection across environmental gradients. Here we present the fully annotated genome of *N. lapillus* generated with Oxford Nanopore Techonology sequencing at ∼37× coverage. The genome assembly is 2.32 Gbp and consists of 2,525 contigs, with an N50 length of 2 Mbp. Repeat annotation identified 2,491 families that cover 67.56% of the genome, which is similar to other gastropods. Despite its large size and high proportion of repeats, the genome is of high quality. Benchmarking Universal Single-Copy Ortholog (BUSCO) analysis revealed a score of 96.8%. Functional annotation of the genome produced 45,848 protein-coding genes with a 96.6% BUSCO score. Genomic resources for mollusks lag behind that of other phyla, perhaps because many of their innate characteristics complicate DNA extraction, sequencing, and assembly. This new *N. lapillus* genome will increase our genomic understanding of the second largest phylum (and the most diverse class within said phylum) and serve as a key resource to advance studies on the organismal biology and population genetics of this iconic species as well as the connection between genomic variation and community-level processes.

## Introduction

The rocky intertidal snail, *Nucella lapillus* (Linnaeus 1758, Fig. 1), is an ectothermic neogastropod that has been a focal organism for studies on organismal performance and rocky shore community dynamics. As a carnivore occupying middle trophic levels of rocky shore food webs, its diet consists mainly of sessile space holders (barnacles, *Semibalanus balanoides* and mussels, *Mytilus edulis*) and its consumption of these prey can strongly shape the structure and dynamics of rocky shore communities (Menge 1976; Hughes and Burrows 1994; Trussell et al. 2003; Bertness et al. 2004). *N. lapillus* is the sole Atlantic species of the genus *Nucella* and descended from a North Pacific ancestor–most likely from its early participation in the trans-arctic interchange of the early Pliocene (7-8 MYA)–and subsequently colonized the Atlantic (Vermeij 1991; Collins et al. 1996). During the last glacial maximum (LGM, around 20,000 YA), *N. lapillus* is thought to have gone locally extinct in the region spanning New England to Canada, followed by post glaciation recolonization via immigrants from eastern Atlantic populations (Wares and Cunningham 2001). Within the Atlantic today, *N. lapillus* is widely distributed, ranging from Greenland to Long Island (USA) in the west and Portugal to Novaya Zemlya (Russia) in the east (Collins et al. 1996; Wares and Cunningham 2001).

**Fig. 1.**
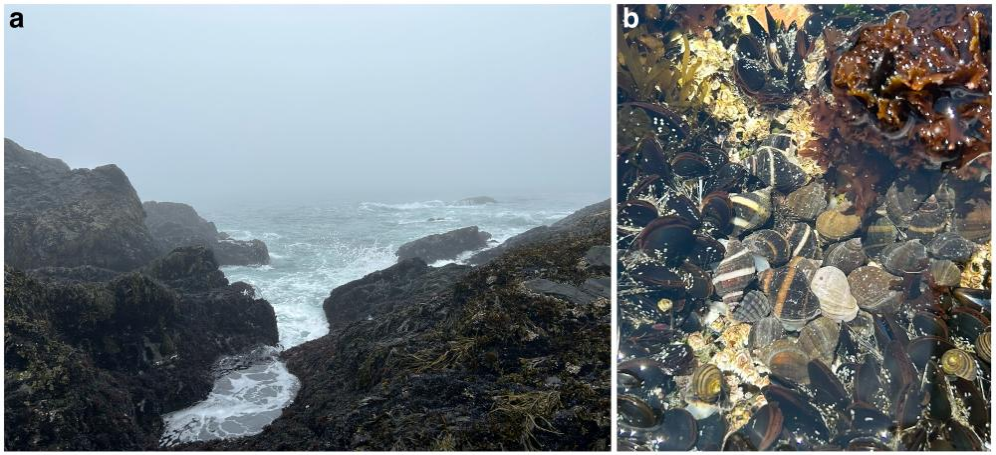
Habitat of *N. lapillus*. a) Habitat landscape of wave-exposed rocky shores in Nahant, Massachusetts where the sample was collected. b) Aggregation of live individuals (Photo credit: M.R.Ford).

*N. lapillus* exhibits substantial geographic variation in a variety of traits that are often shaped by environmental gradients and associated selection pressures (Crothers 1985). Indeed, the large literature on *N. lapillus* has enhanced understanding of the influence of water temperature (Miller 2013, 2014 ), wave exposure (Menge 1978; Etter 1988, 1989), predation risk (Trussell et al. 2003, 2006a, 2006b), and resource availability (Matassa et al. 2016) on organismal and community-level responses as well as evolutionary themes including isolation by environment (Carro et al. 2019), phenotypic evolution (Vermeij 1982), and plasticity (Appleton and Palmer 1988). For example, shell color in *N. lapillus* is partly determined by a selective gradient in physiological stress, with white morphs dominating on thermally stressful sheltered shores and brown/black morphs being more prevalent on cooler, wave-exposed shores (Etter 1988). Bantock and Cockayne (1975) found diploid chromosome numbers (2*n*) to vary between 26 and 27-36 in *N. lapillus* and further research showed that these chromosomal lineages could be discriminated morphologically and genetically (Bantock and Cockayne 1975; García-Souto et al. 2025). The well-established demographic history, remarkable intraspecific variation, and important ecological role of *N. lapillus* make it an excellent species for ecological and evolutionary studies.

The genus *Nucella* contains 6 extant species—5 Pacific species, and *N. lapillus*, the Atlantic species—none of which have published annotated reference genomes (March 2025). Estimated genome size for species in the *Nucella* genus are quite large, ranging from 2.43 to 2.8 picograms (Gregory 2025; Animal Genome Size Database). The genomes of mollusks are generally known to be repeat rich and high in heterozygosity (Gomes-dos-Santos et al. 2020). Both factors, in addition to a larger genome, can lead to fragmented and incomplete assemblies, but this can be remedied by including long-read sequencing at or above 30× and further filtering reads for length (Dominguez Del Angel et al. 2018; Sun et al. 2021). Before assembly begins, the phylum's affinity for polyphenolic proteins and mucopolysaccharides add difficulty to DNA extraction and downstream sequencing (Adema 2021). These characteristics ultimately complicate the sequencing, assembly, and annotation of molluskan genomes.

Nevertheless, we present the assembled and annotated genome of the Atlantic dog whelk, *Nucella lapillus*. This genome was assembled using Oxford Nanopore Technologies (ONT) long reads at ∼37× and annotated using a combination of previously published RNAseq data (Chu et al. 2014) and protein data. The number of published molluskan genomes is lower than other phyla, despite the phylum's ubiquity (comprising the second largest animal phylum) and ecological and commercial importance (Gomes-dos-Santos et al. 2020; Sun et al. 2021). In this study we successfully extracted and sequenced high molecular weight (HMW) DNA from foot tissue of *N. lapillus*. This new genomic resource, leveraged with ecological and evolutionary studies, will be essential to building more robust predictions regarding the impacts of environmental change on rocky shore communities.

## Materials and methods

### Sample collection and DNA isolation

An adult *N. lapillus* was collected from the wave-exposed shores of Nahant, MA in May 2024 (42.419732, −70.902171). HMW DNA was extracted from the foot via the CTAB method (1.4 M NaCL and 2% CTAB), followed by 3 chloroform reactions. The full extraction protocol is available on the project GitHub repository (https://github.com/meghanclownfish/Nucella-lapillus-genome/tree/main/1_extraction). The HMW DNA was precipitated in ethanol (EtOH) and resuspended in TE buffer. We further purified the sample using a Genomic DNA Clean and Concentrator kit (gDCC-10, ZYMO Research, Irvine, CA, USA) per manufacturer’s instructions. Sample quality and concentration were assessed by running 2 μL of the sample on Nanodrop (Thermo Fisher Scientific, Singapore). A sample was deemed ready for sequencing if the 260/280 ratio was ∼1.9 and the 260/230 ratio fell between 2.0 and 2.2, following Sun et al. 2020.

### ONT library preparation and sequencing

We sequenced *N. lapillus* long reads using ONT platforms. Libraries were prepared with the ONT Ligation sequencing kit (SQK-LSK114, ONT, Oxford, UK) and NEBNext Companion Module (E7180S NEB). Standard manufacturer's protocol was implemented with the following exceptions: incubation time for each step was increased to 30 min; EtOH was chilled before use; and the volume used per wash was increased to 500 μL. Additionally, the final elution was performed at 37 °C for 30 min to increase recovery of longer DNA fragments. Because residual contaminants from the foot tissue can clog flow cell pores, DNA input concentration varied between flow cells and libraries. Concentrations for each load can be found in Supplementary Table 1 in Supplementary File 1. PromethION flow cells (FLO-PRO114M) were primed and loaded per the standard manufacturer's protocol. To increase the yield of each flow cell, runs were paused and flushed using the EXP-WSH004 (ONT) kit and reloaded. High quality base calling was performed with Dorado 0.7.1 (ONT).

### Genome size and heterozygosity

We estimated genome size using JELLYFISH v2.2.10 to count canonical 41-mers from high quality ONT reads (min quality: 5) and computed a histogram of k-mer occurrence (Marçais and Kingsford 2011). This histogram was used to estimate heterozygosity with GenomeScope 2.0 (Ranallo-Benavidez et al. 2020).

### Genome assembly

Adapter sequences were removed with PORECHOP (v0.2.4, https://github.com/rrwick/Porechop). Reads from all flow cells were combined into 1 fastq file and filtered for assembly (min length: 2 Kb) or k-mer counting (min quality: 5) using SeqKit (v2.9.0, Shen et al. 2024). Hifiasm (0.25.0-r726) assembled the reads into haplotype-resolved initial contigs (Cheng et al. 2024, flags: –ont). We visually examined and filtered contigs using BlobTools2 (v4.4.0, Challis et al. 2020). Possible contamination was removed based on DIAMOND (Buchfink et al. 2015) matches to the UniProt database (The UniProt Consortium 2025). Any contigs of non-metazoan origin (e.g. algal and bacterial), or shorter than 15 Kb were removed. To remove the mitochondrial genome, we used the existing mitogenome for *N. lapillus* as the query in a blastn search against the genome on the Galaxy platform (Altschul et al. 1990; García-Souto et al. 2024; The Galaxy Community 2024). BUSCO was used to monitor the assembly at each step with the odb10 metazoan dataset (v5.8.2, Manni et al. 2021). The Quality Assessment Tool for Genome Assemblies, or QUAST (Gurevich et al. 2013), was used on the Galaxy platform (The Galaxy Community 2024) to assess the quality of the final assembly. Before moving on to annotation, FCS-adapter and FCS-GX were used as a final check for adapter and biological contamination in the genome (Astashyn et al. 2024).

### Genome annotation

Repeat content in the *N. lapillus* genome was detected *ab initio* with RepeatModler (v2.0.6, Flynn et al. 2020) via RECON (v1.08) and RepeatScout (v1.0.7). The repeat library identified by 5 rounds of RepeatModeler was used as input for RepeatMasker. RepeatMasker (v4.1.7-p1, https://www.repeatmasker.org/cgi-bin/WEBRepeatMasker) soft-masked the repetitive elements found in the genome using RMBlast (v2.14.1+). Published RNAseq data from *N. lapillus* was downloaded from the NCBI Sequence Read Archive (SRA- SRX357400). TrimGalore (v0.6.10, https://github.com/FelixKrueger/TrimGalore), along with Cutadapt (v5.0, https://github.com/marcelm/cutadapt) and FastQC (v0.11.9, https://www.bioinformatics.babraham.ac.uk/projects/fastqc), removed adapters and filtered reads by quality with default settings. Cleaned reads were then mapped to the soft-masked genome with HISAT2 (v2.2.0, Kim et al. 2019) and merged into a single file.

BRAKER3 in ETP-mode used the merged RNAseq file as well as metazoan protein data (OrthoDB v.12, Tegenfeldt et al. 2025) and molluskan sequences pulled from NCBI's protein database (March 2025) as evidence for gene model predictions on the soft-masked genome (Gabriel et al. 2024). TSEBRA (Gabriel et al. 2021) was used to combine the gene sets created by BRAKER3, enforcing the Augustus set (Stanke et al. 2008). We removed single exon genes and incomplete gene models and retained only the longest isoforms with TSEBRA (Gabriel et al. 2021), AGAT (v1.4.2, Dainat 2022) and BRAKER3 (Gabriel et al. 2024) scripts, respectively. The composition of the filtered set of gene models was evaluated with BUSCO (odb10 metazoan, protein mode). Functional annotation of the final gene sets for *N. lapillus* was performed with Interproscan (v5.73, Jones et al. 2014) and Funannotate (Palmer and Stajich 2020). OMArk was used to assess the quality of the produced proteome and compare it to published lophotrocozonan and gastropod proteomes downloaded from NCBI in Febuary 2022 (Nevers et al. 2025).

## Results and discussion

### Genome size and heterozygosity

**Fig. 2.**
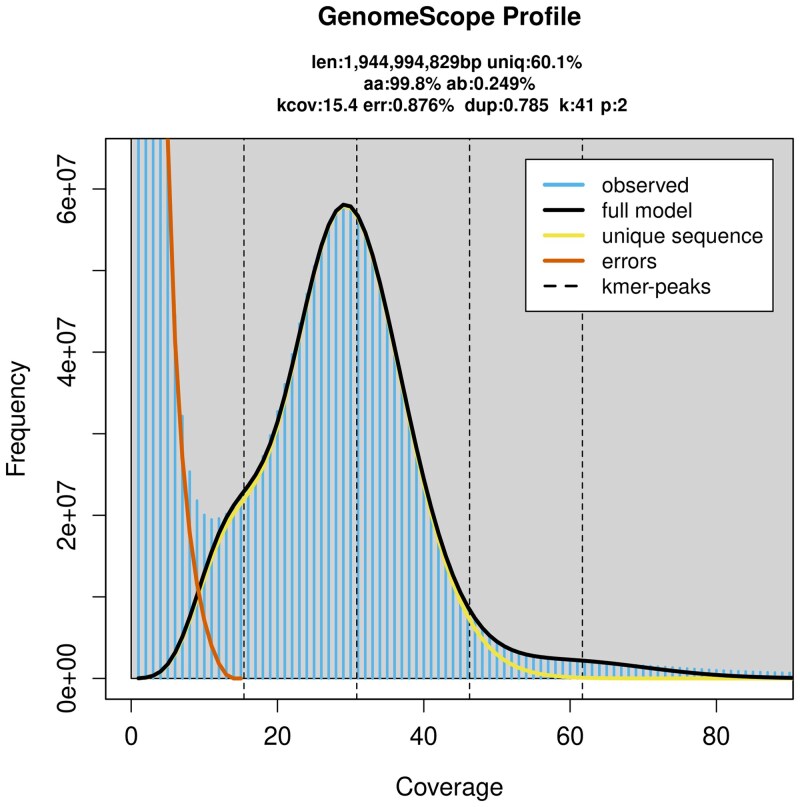
GenomeScope2 k-mer profile plot based on 41-mer for *N. lapillus*.

ONT reads of Q5 or higher were used to estimate genome size and heterozygosity. The 41-mer based genome size estimate provided by JELLYFISH and GenomeScope 2 is ∼1.9 Gbp, which is lower than expected (Fig. 2). In the reference individual, heterozygosity levels were estimated to be 0.249%, which is lower than other gastropods. For example, the heterozygosity of the grove snail (*Cepaea nemoralis*) is 1.42% (Saenko et al. 2021), 1.09% for the land snail *Candidula unifasciata* (Chueca et al. 2021), and 1.41% for the caenogastropod *Rapana venosa* (Song et al. 2023). This lower observed heterozygosity in *N. lapillus* may be a result of smaller effective population sizes due to poor dispersal, or a lingering effect of the local extinction during the LGM. The difference in the estimated and assembled genome size is likely driven by the high-frequency of repeats. Specifically, high-frequency repeats are hard to capture and lead to an overall underestimation of genome size (e.g. Edwards et al. 2018; Saenko et al. 2021). Previous estimates using Feulgen Densitometry and Bulk Fluorometric Assay place the genome of *N. lapillus* at 2.60–2.80 picograms (Gregory 2025; Animal Genome Size Database). This value is much closer to the genome assembled here.

### ONT library preparation and sequencing

Residual polyphenolic proteins and mucopolysaccharides can cause clogging of ONT pores, failed sequencing, and unreliable results (Personal observation, Fuchs et al. 2024). We found that neither freezing tissues in liquid nitrogen, nor doing a phenol/chloroform clean-up on extracted HMW DNA was sufficient; both resulted in clogged pores and a failed sequencing run. Extracting HMW DNA with the CTAB method did resolve the clogged pores issue (the extraction protocol can be found on the project GitHub repository https://github.com/meghanclownfish/Nucella-lapillus-genome/tree/main/1_extraction). However, after loading, the pores available decreased exponentially, down to about 100 after ∼12 h. This behavior was also noted in Chueca et al. (2021). We found that loading less DNA (lower library concentration), and reloading the flow cell multiple times, increased overall output.

### Genome assembly

In total, 6 ONT PromethION flow cells were used to obtain 103,553,219,099 bp of raw data with an N50 of 6.77 Kb, average length of 2.99 Kb and a median length of 1.35 Kb. The raw data was then filtered and only reads of 2 Kb or greater in length were used for assembly to increase genome contiguity (Sun et al. 2021), resulting in 13,173,883 ONT reads (∼86 Gb). The assembly reads had an N50 of 8.72 Kb, an average length of 6.50 Kb, and a median legth of 4.30 Kb. Of the assembly reads, 78.45% of the bases exhibited a quality score higher than 20. The draft assembly generated by Hifiasm spanned 2.332 Gbp across 2,794 contigs with roughly 37× coverage. Quality filtering and decontamination of the assembly removed any contigs not of metazoan origin (94 contigs), shorter than 15 Kb (172 contigs), or mitochondrial (3 contigs). We did not need to polish or run purge_dups on the assembly because Hifiasm includes each step. After filtering, the final assembly spanned 2.322 Gbp across 2,525 contigs and exhibited high completeness [96.8% (S: 87.6%, D: 9.1%), F: 0.6%, M: 2.6%, *n*: 954, Fig. 3]. The longest contig was 10.84 Mbp and the assembly had an L50 of 353 (Table 1). The final check with FCS revealed no biological or adapter contamination.

**Fig. 3.**
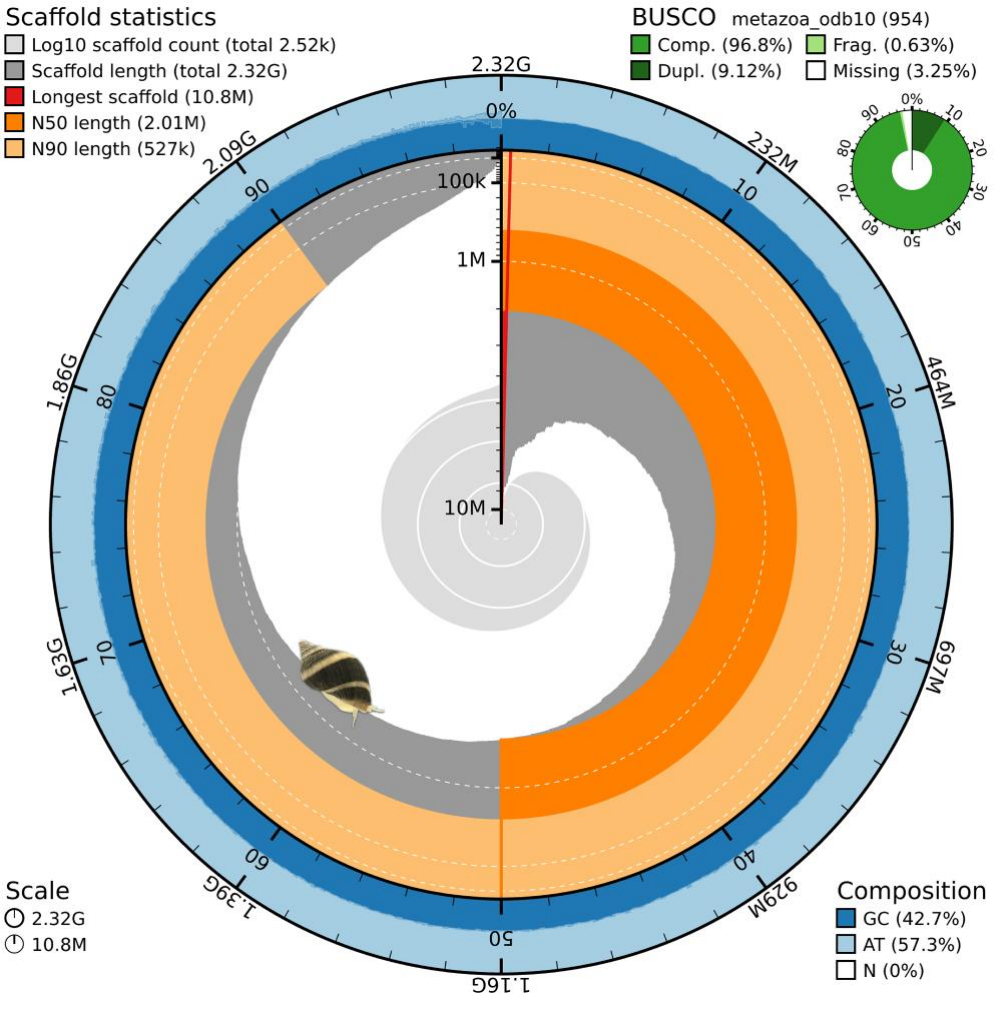
Snail plot of *N. lapillus* genome showing contiguity and completeness statistics.

**Table 1.** *Nucella lapillus* genome assembly and annotation statistics.

Genome assembly summary	
Total length (Gbp)	2.322
Contig N50 (Mbp)	2.014
Longest contig (Mbp)	10.846
Number of contigs	2,525
L50	353
GC level	42.7%
BUSCO (v5.8.2, metazoa_odb10, *n* = 954)	
Genome	
Complete [single/duplicated]	96.8% [87.6%/9.1%]
Fragmented	0.6%
Missing	2.6%
Annotation	
Complete [single/duplicated]	96.6% [87.9%/8.7%]
Fragmented	2.0%
Missing	1.4%

### Genome annotation

RepeatModeler and RepeatMasker identified 2,510 families, spanning 67.88% of the genome (Table 2). The most common type of repeat element identified were long interspersed nuclear elements, or LINEs, and they comprised 21.19% (492,065,374 bp) of the genome. The repeat content and identity of *N. lapillus* are comparable with other gastropods. The genome of the Neogastropod *Fulgoraria chinoi* is 53.97% repeat content, with 15.21% attributed to LINEs (H. Wang et al. 2024 ) and the genome of *C. unifasciata* has a total repeat content of 61.10%, LINEs making up 25.03% (Chueca et al. 2021).

**Table 2.** Repetative elements in the *N. lapillus* genome.

			Number of elements	Length occupied (bp)	Percentage of sequence
Retroemelents			2,691,946	781,451,738	33.65
	SINEs:		277,189	36,044,329	1.55
	Penelope:		75,176	6,881,310	0.30
	LINEs:		1,764,911	492,065,374	21.19
		CRE/SLACS	0	0	0.00
		L2/CR1/Rex	1,003,682	297,219,447	12.80
		R1/LOA/Jockey	219,244	40,742,518	1.75
		R2/R4/NeSL	1469	275,464	0.01
		RTE/Bov-B	374,382	109,651,045	4.72
		L1/CIN4	1259	345,377	0.01
	LTR elements:		649,846	253,342,035	10.91
		BEL/Pao	1589	2,171,549	0.09
		Ty1/Copia	2707	4,231,137	0.18
		Gypsy/DIRS1	525,016	217,960,382	9.39
		Retroviral	0	0	0.00
DNA transposons			595,702	104,628,022	4.51
	hobo-Activator		76,011	13,646,657	0.59
	Tc1-IS630-Pogo		21,791	6,215,748	0.27
	En-Spm		0	0	0.00
	MULE-MuDR		197,628	14,998,276	0.65
	PiggyBac		4689	6302263	0.27
	Tourist/Harbinger		1835	747,486	0.03
	Other (Mirage, P-element, Transib)		121,133	25,322,104	1.09
Rolling-circles			9238	2,506,695	0.11
Unclassified:			2,278,203	305,473,454	13.15
Total interspersed repeats:				1,198,434,524	51.61
Small RNA:			553,661	118,589,147	5.11
Satellites:			161	21508	0.00
Simple repeats:			2,643,129	26,463,0731	11.40
Low complexity:			170,158	16,041,084	0.69

HISAT2 aligned 89.84% of the RNAseq data to the soft-masked reference genome. We used this data along with a custom protein database to annotate the genome with BRAKER3. TSEBRA was used to combine the gene sets created by BRAKER3, enforcing the set created by Augustus. We then filtered the genes by removing single exon genes and incomplete gene models, and keeping only the longest of each isoform. This resulted in 67,815 predicted genes. Funnannotate and Interproscan identified 45,848 protien-coding genes via gene family and/or similarity search. The remaining 32% of the genes were unannotated. This proportion is normal for lophotrochozoans (Supplementary Fig. 2 in Supplementary File 1) and the number of functionally annotated genes is comparable to other marine and land snails (Schell et al. 2017; Lan et al. 2021; Y.-S. Wang et al. 2024). The annotation was then assessed with BUSCO in protein mode and indicated high quality and completeness [Table 1, 96.6% (S: 87.9%, D: 8.7%), F: 2.0%, M: 1.4%, *n*: 954].

Given the elevated number of duplicate BUSCO genes and large number of gene annotations, it is possible that the assembly contains uncollapsed heterozygosity. Default Hifiasm haplotig purging was used in the assembly based on the low estimated heterozygosity (0.249%, Fig. 2). In other published caenogastropod genomes, duplicate BUSCO genes range from 11% in *Conus betulinus* to 5.5% in *Rapana venosa* (Peng et al. 2021 ; Song et al. 2023). Hence, although the duplicate BUSCO genes we observed are within this range, we wanted to make readers aware that uncollapsed heterozygosity may be present in this assembly.

**Fig. 4.**
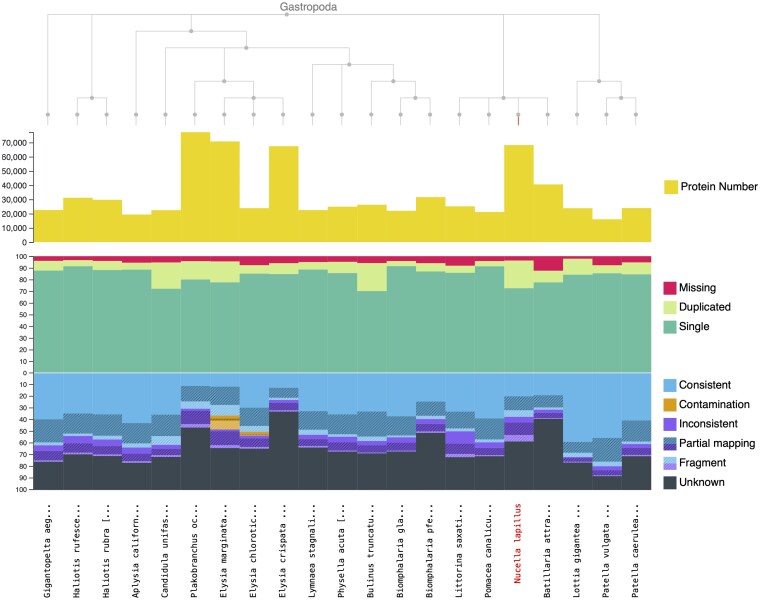
OMArk plot of *N. lapillus* (fifth from the right) proteome compared with other gastropods.

OMARk is a software package that uses alignment-free sequence comparisons between a proteome of interest and precalculated gene families, it then assess completeness, consistency, and contamination (Nevers et al. 2025, https://omark.omabrowser.org). OMArk revealed the proteome had no contamination, and its gene models are like that of other gastropods (Fig. 4, Supplementary Fig. 1 in Supplementary File 1). *N. lapillus* clusters within its subclass, Caenogastropoda. Other species present in this OMARk cluster are *Littorina saxatilis*, *Pomacea canaliculata*, and *Batillaria attramentaria* (Fig. 4).

## Conclusion

Here we report the annotated genome of the Atlantic dog whelk *Nucella lapillus*. This ∼2.3 Gbp assembly spans 2,525 contigs with an N50 of 2.01 Mbp and 37x coverage. To our knowledge, this is the first published and annotated genome for the *Nucella* genus. Despite the abundance of repeats (over half the genome), and its large size, the assembly and annotation are of high quality and completeness. Regarding the “sticky molluskan situation”, we found that extraction with the CTAB method was sufficient for ONT sequencing, and that a lower library concentration split into multiple loads increased overall yield. This genomic resource will enable studies of how geographic history, gene flow, founder events, and environmental gradients shape ecological and evolutionary dynamics on rocky shores. In addition, it will enhance studies seeking to link genomic variation with organismal performance and community-level (i.e. “genes to ecosystems”) processes under various dimensions of environmental change.

## Supplementary Material

jkaf182_Supplementary_Data

## Data Availability

This Whole Genome Shotgun project has been deposited at DDBJ/ENA/GenBank under the accession JBNHDL000000000. The version described in this paper is version JBNHDL010000000. Data generated in this study are available under NCBI BioProject PRJNA1238877. The RNAseq data used to annotate the assembly can be found at SRA-SRX357400. Information on the protocol used for DNA extraction and commands used to assemble and annotate the genome can be found on the project GitHub: https://github.com/meghanclownfish/Nucella-lapillus-genome. Supplemental material available at G3 online.
