# Correction to: Annotated genome of the Atlantic dog whelk, *Nucella lapillus*

**DOI:** 10.1093/g3journal/jkag102

**Published:** 2026-05-06

This is a correction to: Meghan R Ford, Steven V Vollmer, Geoffrey C Trussell, Annotated genome of the Atlantic dog whelk, *Nucella lapillus*, *G3 Genes|Genomes|Genetics*, Volume 15, Issue 10, October 2025, jkaf182, https://doi.org/10.1093/g3journal/jkaf182

In the originally published version of this manuscript, a reference was missing from the **Literature Cited** list:

“Adema CM. 2021. Sticky problems: extraction of nucleic acids from molluscs. Philosophical Transactions of the Royal Society B: Biological Sciences. 376(1825):20200162”. https://doi.org/10.1098/rstb.2020.0162.

This has been added to the **Literature Cited** section in its correct alphabetical place.

