# Supplementary material for: Annotated genome of the Atlantic dog whelk, Nucella lapillus
Source: G3 (Bethesda). 2025 Aug 9;15(10):jkaf182. doi: 10.1093/g3journal/jkaf182 (PMC12506669; doi:10.1093/g3journal/jkaf182)
Supplement: jkaf182_Supplementary_Data [file jkaf182_supplementary_data.docx]

**Supplementary Tables**

Supplementary Table 1: Flow cell input library concentrations and output in base pairs.

| Flow cell library concentration (ng/ μL) and loads | | | | | |
| --- | --- | --- | --- | --- | --- |
| Flow cell | Load I | Load II | Load III | Load IV | Load V |
| 1 (6/30/24) * | 12 | x | x | x | x |
| 2 (7/10/24) | 26.8 | 33.5 | x | x | x |
| 3 (7/16/24) | 13.5 | 6.75 | 6.75 | x | x |
| 4 (8/26/24) | 6.06 | 6.06 | 6.06 | x | x |
| 5 (9/22/24) | 7.4 | 7.4 | 4.6 | 4.6 | 4.6 |
| 6 (2/4/25) | 16.38 | 10 | 10 | x | x |
| Raw flow cell output summary | | | | | |
|  | Output (bp) | N50 | Max length | Q20 (%) | GC (%) |
| 1* | 5,336,085,290 | 5,612 | 655,757 | 45.57 | 40.67 |
| 2 | 11,761,809,163 | 8,042 | 475,726 | 79.26 | 42.41 |
| 3 | 25,332,111,648 | 7,089 | 924,327 | 81.14 | 42.38 |
| 4 | 20,574,027,030 | 7,487 | 805,927 | 75.53 | 42.23 |
| 5 | 18,104,849,907 | 5,934 | 675,098 | 71.45 | 42.21 |
| 6 | 22,780,498,520 | 5,786 | 1,429,348 | 87.37 | 42.5 |

*Flow cell 1 did not pass QC because of low initial pore availability. We ran a library on it anyway to see if the clogged pore problem was fixed with CTAB.

**Supplementary Figures**


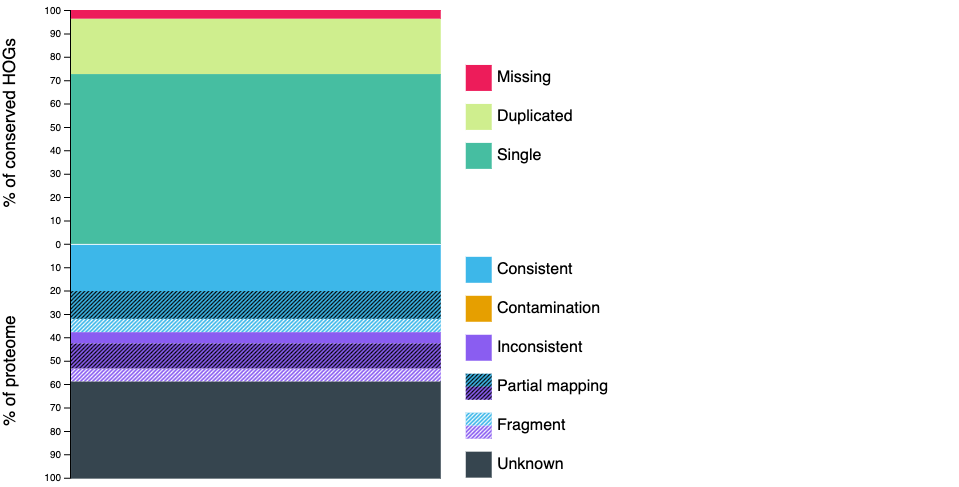


Supplementary Figure 1. OMArk completeness and whole proteome assessment results for *N. lapillus*.


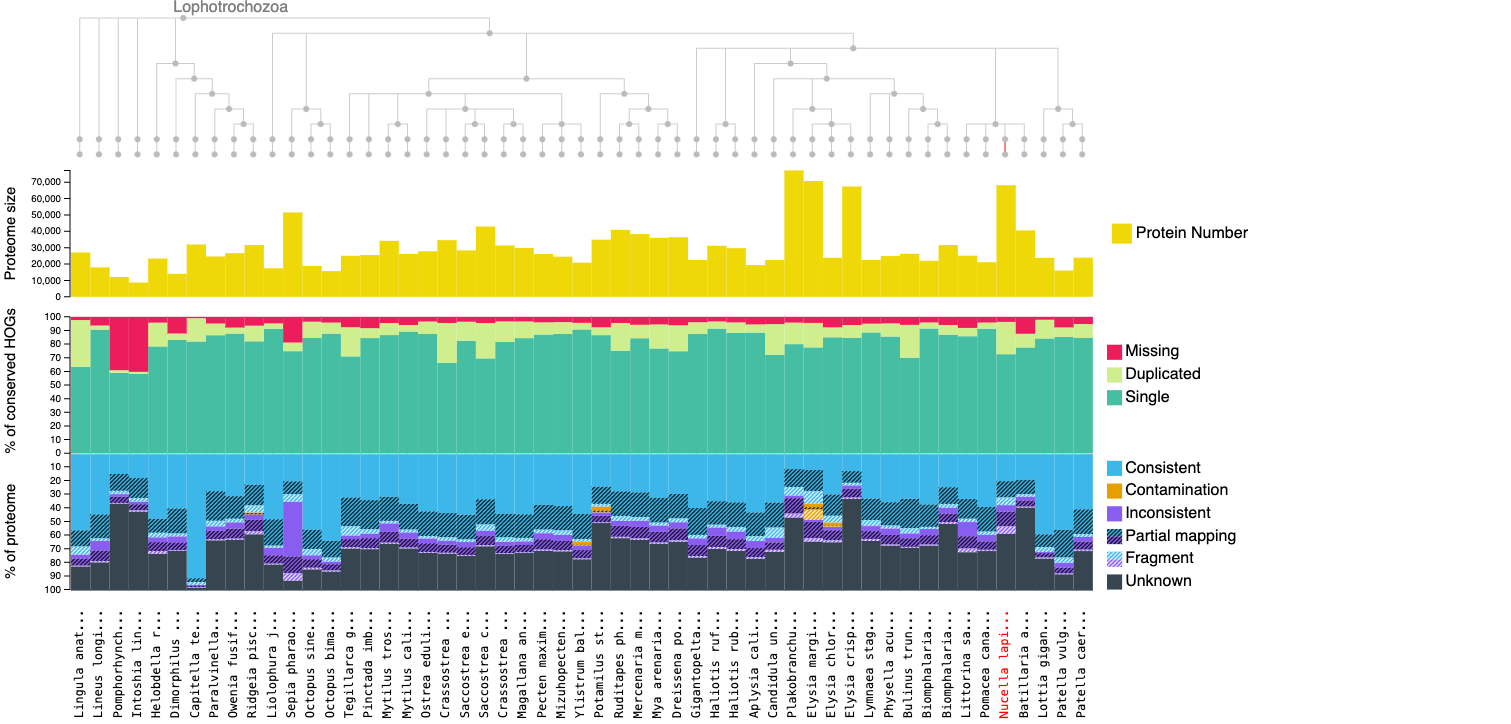


Supplementary Figure 2. OMArk completeness and whole proteome assessment results for *N. lapillus* (in red) among other lophotrochozoans.
